# Effect of Cementitious Capillary Crystalline Waterproof Material on the Resistance of Concrete to Sulfate Erosion

**DOI:** 10.3390/ma18204659

**Published:** 2025-10-10

**Authors:** Guangchuan Fu, Ke Tang, Dan Zheng, Bin Zhao, Pengfei Li, Guoyou Yao, Xinxin Li

**Affiliations:** 1 Chongqing Water Conservancy Harbor and Channel Construction Group Co., Ltd., Chongqing 400020, China; lxctgulx@hotmail.com; 2 School of River and Ocean Engineering, Chongqing Jiaotong University, Chongqing 400074, Chinazhengdan@cqjtu.edu.cn (D.Z.);; 3 China Railway First Group Co., Ltd., Xi’an 710054, China; 4 Suzhou Guardex New Material Technology Co., Ltd., Suzhou 210500, China

**Keywords:** cementitious capillary crystalline waterproof material, sulfate erosion, concrete, compressive strength, microstructure

## Abstract

Concrete structures are vulnerable to sulfate attacks during their service life, as sulfate ions react with cement hydration products to form expansive phases, generating internal stresses that cause mechanical degradation. In this study, a cementitious capillary crystalline waterproofing material (CCCW) was incorporated into concrete to mitigate sulfate ingress and enhance sulfate resistance. The evolution of compressive strength, ultrasonic pulse velocity, dynamic elastic modulus, and the microstructure of concrete was investigated in sulfate-exposed concretes with varying CCCW dosages and strength grades; the sulfate ion concentration profiles were also analyzed. The results indicate that the enhancement effect of CCCW on sulfate resistance declines progressively with increasing concrete strength. The formation of calcium silicate hydrate and calcium carbonate fills the pores of concrete, hindering the intrusion of sulfate solution. Moreover, the self-healing effect of concrete further inhibits the diffusion of sulfate ions through cracks, improving the sulfate resistance of concrete. These findings provide critical insights and practical guidance for improving concrete resistance to sulfate-induced deterioration.

## 1. Introduction

Concrete structures such as dams, reservoirs, ports and bridges are extremely susceptible to sulfate erosion during their service life [1]. Sulfate attacks are one of the key factors that lead to concrete deterioration. The corrosive media carried by sulfates gradually diffuse into the interior of the concrete. Sulfate ions react with cement hydration products to form expansive crystals, causing the concrete to expand and crack. Ultimately, a damaged layer forms and prompting cracks and surface spalling in concrete, weakening the structural integrity and bearing capacity [2,3,4,5]. To reduce sulfate attacks of concrete, many researchers have enhanced concrete performance by introducing basalt fiber and polypropylene fiber into concrete [6,7,8,9]. In recent years, significant progress has been made in the research and development of permeable crystallization materials, mainly focusing on the crack self-healing performance. Zhang et al. [10] prepared CCCW with different dosages and studied the crack self-healing performance. The test showed that CCCW can effectively promote the recovery of concrete strength to the uncracked state. Lee et al. [11] used CCCW to repair prefabricated cracks and verified the self-healing performance of CCCW through Brazilian splitting tests and numerical simulation methods. Sisomphon et al. [12] explored the self-healing performance of CCCW on concrete specimens under different environments. The test showed that the calcium carbonate generated at the crack can exhibit excellent watertightness. CCCW, as a new waterproof building material, was first invented by a German scientist to solve the problem of cement leakage in ships [13,14,15,16,17]. CCCW is primarily made by mixing raw materials such as ordinary Portland cement and quartz sand with specially formulated active chemicals [18,19,20]. The composition, functional components, selection of the main base material, and preparation method of the permeable crystallization material significantly affect the material’s waterproofness, self-healing ability, and durability [21,22,23]. The active chemicals in CCCW have strong permeability and can penetrate into the pores and capillaries within concrete to form a dense crystalline network, improving the cement-based material’s resistance to sulfate attack, while also enhancing the concrete’s structural strength and protecting steel from corrosion [18].

Long-term exposure of concrete to a sulfate environment will lead to sulfate corrosion, resulting in the deterioration of various mechanical properties of concrete. Bassuoni and Nehdi [24] used sodium sulfate solution to corrode self-compacting concrete. The study found that the mass and dynamic elastic modulus of the corroded concrete increased with the increase in corrosion time. Güneyisi et al. [25] conducted a compressive test on concrete corroded by sodium sulfate. The test showed that the compressive strength of concrete gradually decreased after sulfate corrosion, with the maximum reduction reaching 40%. Yao et al. [26] found that the compressive strength of concrete decreased significantly after 120 days of sulfate corrosion. A model relationship between ultrasonic wave velocity and compressive strength under sulfate corrosion was established, and it was found that the wave velocity and compressive strength were highly correlated. The above studies show that the degradation of static and dynamic properties under sulfate corrosion is crucial for durability assessment and structural safety. The sulfate attack damage mechanism of concrete involves the coupling of multiple factors, and its complexity stems from the interaction between environmental factors and material properties. In this attack process, the sulfate ion diffusion coefficient can quantitatively characterize the migration rate of the corrosive medium in the concrete pore structure and its temporal and spatial distribution. Therefore, researchers have proposed different models to describe the diffusion characteristics of sulfate ions. The sulfate ion diffusion coefficient is the core parameter that characterizes its migration rate in concrete. The smaller the diffusion coefficient, the higher the concrete density, the stronger the impermeability, and the slower the sulfate attack process [27]. Jin et al. [28] constructed a time-varying diffusion coefficient prediction model based on the non-steady-state diffusion theory. Gospodinov et al. [29] systematically measured the porosity evolution law and the sulfate ion content involved in the reaction of specimens with different attack cycles, and established an effective diffusion coefficient calculation model that takes into account the chemical reaction consumption effect. Sun et al. [30] established a diffusion coefficient prediction equation under the influence of multiple factors such as diffusion coefficient and water-cement ratio, solution concentration, stress ratio, and curing time. The establishment of these theoretical models not only reveals the kinetic nature of sulfate transport, but also provides an important theoretical basis for concrete durability design and life prediction. The sulfate ion diffusion coefficient in concrete is closely related to the CCCW dosage, concrete strength grade, and erosion time [31,32]. However, existing research has largely focused on sulfate attack behavior at a single strength grade or under a specific dosage. The influence of CCCW content and concrete strength on the mechanical properties and the diffusion behavior of sulfate ions in sulfate-eroded concrete remains limited.

Therefore, this study aims to investigate the sulfate ion attack process and analyze the diffusion process in concretes with different strength grades and varying CCCW dosages. The study examines the variation in concrete mass, compressive strength, ultrasonic velocity, dynamic elastic modulus, and microstructure, as well as the sulfate ion concentration profiles. The findings provide a deeper understanding of the transport behavior and reaction mechanisms of sulfate in CCCW-containing concrete.

## 2. Experimental Materials and Methods

### 2.1. Experimental Materials

The concretes with designed 28-day compressive strengths of C20(L), C30(M), and C45(N) were selected for analysis in this study. The primary raw materials used were ordinary Portland cement, fine aggregate (river sand), coarse aggregate, mixing water, and CCCW. The cement used was Portland cement of grade 42.5 (P.O. 42.5), while the fine aggregate consisted of medium-sized natural river sand with an apparent density of 2.63 g/cm^3^, a bulk density of 1.53 g/cm^3^, and a fineness modulus of 2.62. The CCCW was an internally admixed cement-based permeable crystalline waterproofing agent developed by Suzhou Jiagushi New Materials Technology Co., Ltd. (Suzhou, China). The components are composed of Portland cement, quartz sand, and a specialized activator to form the CCCW material. CCCW chemically reacts with the moisture in cementitious composites, generating insoluble crystalline products. When cracks or pores appear in concrete, the active constituents in the crystalline material react with water to form additional crystalline phases. These crystals grow within the cracks and pores, filling defects and repairing internal or surface damage in the concrete. In accordance with the manufacturer’s recommendations, CCCW contents were 0, 3, 6, 9, and 12 kg/m^3^. Concrete specimens were cast and allowed to stand for 24 h after pouring to facilitate initial setting before demolding. After demolding, specimens were cured in a curing room until reaching 28 days of age. Consequently, a total of 11 mix proportions were designed for this study and are presented in Table 1. Here, L12 denotes a C20 concrete containing 12 kg/m^3^ of CCCW.

Specimens were cast in a standard mold measuring 100 mm × 100 mm × 100 mm. For each mix ratio, 18 cubic specimens were prepared to monitor mass loss, test compressive strength, analysis ultrasonic propagation, and determine sulfate ion concentration. In addition, a 100 mm × 100 mm × 400 mm rectangular specimen was cast for dynamic elastic modulus testing. The exact number of specimens planned for each condition is presented in Table 2.

### 2.2. Experimental Process

**Figure 1:**
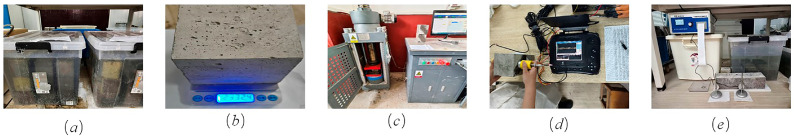
(**a**) Na_2_SO_4_ solution soak; (**b**) weighing of corroded concrete specimens; (**c**) compression test equipment; (**d**) ultrasonic velocity; (**e**) dynamic elastic modulus of concrete.

Sulfate type has a significant influence on the sulfate attack test of concrete. Most studies use sodium sulfate and magnesium sulfate solutions as attack media. Experimental results indicate that magnesium sulfate solution exhibits a composite corrosion effect on concrete. To eliminate the interference from Mg^2+^ ions, this study employed a sodium sulfate solution to establish a single-factor attack environment, with the sodium sulfate concentration controlled at 10% (Figure 1a). The mass-change test was conducted using an electronic balance to measure the mass of concrete before and after exposure, and the mass-change rate was calculated (Figure 1b). The compressive strength of the corroded concrete was measured with a loading rate of 0.3–0.5 MPa/s (Figure 1c). Ultrasonic tests were performed with a non-metallic ultrasonic detector to measure the ultrasonic wave velocity of concrete specimens at different attack times (Figure 1d). The dynamic elastic modulus was measured using a dynamic elastic modulus tester (by Beijing Kangluda Experimental Instrument Co., Ltd. Beijing, China) for specimens at varying attack times (Figure 1e). During testing, specimens were removed from the immersion solution, dried, and then their mass and dimensions were measured. When setting up the measurement equipment, the transmitter probe was gently touched to the mid-perpendicular line of the specimen’s long axis, and the receiver was positioned 5 mm from the bottom edge. A small amount of coupling agent was applied to the probe and concrete.

**Figure 2:**
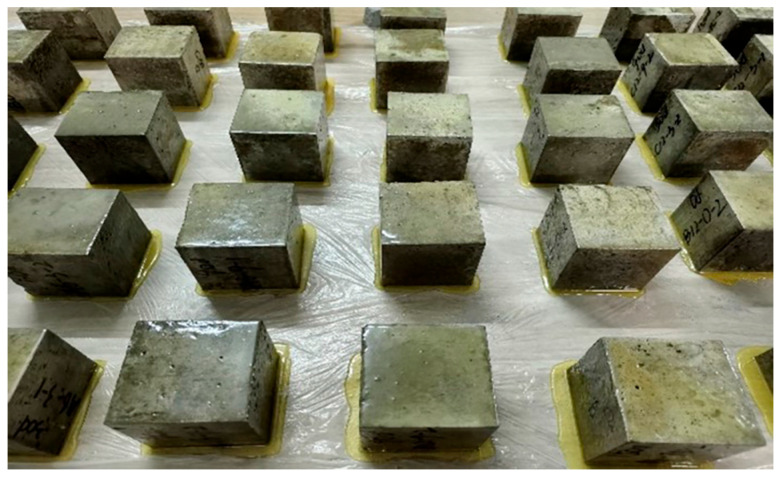
Five faces of the test specimens coated with epoxy resin.

After completing 28 days of standard curing, the specimens were kept in the laboratory for approximately six months and then immersed in a Na_2_SO_4_ solution (pH 6, 10% *w*/*w*) at 23 °C for corrosion testing [33,34,35]. The solution was refreshed every two weeks to maintain a concentration of 10%. For the one-dimensional corrosion test group, as illustrated in Figure 2, a single exposure face was designated, while the remaining five faces were sealed with a two-component epoxy resin (mixed at a 1:1 mass ratio). The exposed surface is the sodium sulfate solution erosion surface.

**Figure 3:**
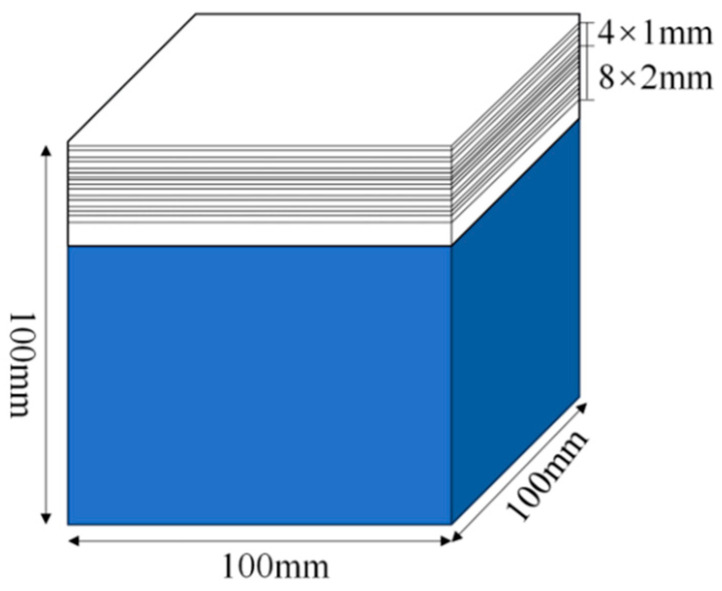
Schematic diagram of stratified sampling.

**Figure 4:**
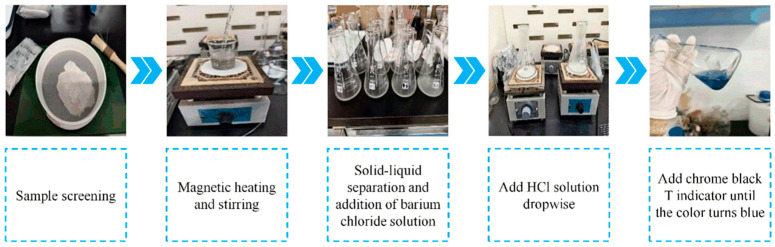
Schematic diagram of the steps for EDTA titration.

Owing to its favorable balance of simplicity, speed, and sufficient precision, EDTA complexometric titration was adopted in this study for sulfate ion determination. Before analysis, specimens exposed for the designed durations were removed from the immersion solution, and the encapsulating epoxy resin was ground away to access the internal concrete matrix. Layered sampling was performed with a DRB-H3 profile grinder, starting at the exposed surface and progressing along the diffusion gradient. To capture near-surface variations, samples were collected at 1 mm intervals from 0 to 4 mm depth, and at 2 mm intervals from 4 to 20 mm depth. The layered sampling protocol is shown in Figure 3, and the detailed EDTA titration procedure is presented in Figure 4.

During the tests, BaCl_2_ reacts with sulfate ions (SO_4_^2−^) in the sample to form insoluble BaSO_4_. After the reaction, residual Ba^2+^ remains in solution. In an alkaline medium (pH ≈ 10) containing Mg^2+^, a complexation reaction occurs between the chrome black T indicator and the metal ions, producing a purple-red solution. Since EDTA has a higher complexing strength than chrome black T, it continuously chelates the metal ions to form a chelate (MY), turning the solution blue. By titrating the remaining Ba^2+^ with a standard solution of the acid C_10_H_16_N_2_O_8_, the sulfate concentration can be indirectly determined. Based on the EDTA titration results, the molar concentration of sulfate ions can be calculated via Equation (1), and the mass fraction of sulfate ions in concrete can be obtained via Equation (2).

(1) $$c\left(SO_{4}^{2-}\right)=\frac{c\left(BaCl_{2}\right)\cdot V_{BaCl_{2}}-c\left(EDTA\right)\cdot \left(V_{2}-V_{1}\right)}{10}$$

(2) $$\omega =\frac{c\cdot V\cdot M}{m}\times 100\%$$

where *c* is the solution concentration (mol/L), *V* is the solution volume (mL), *ω* is the mass fraction of sulfate ions, *M* is the molar mass of sulfate ions (g/mol) and *m* is the mass of concrete powder (g).

To verify the accuracy of the method, considering the low sulfate content (SO_4_^2−^) in concrete, a 10% Na_2_SO_4_ sample was tested, and a 0.005 mol/L Na_2_SO_4_ standard solution was prepared simultaneously. Exactly weighed 0.178 g of anhydrous Na_2_SO_4_ was dissolved in a water bath and diluted to 250 mL with ultrapure water. From this solution, 10 mL were taken and reacted with 10 mL of 0.02 mol/L BaCl_2_, allowed to stand for 10 min, then 1 drop of hydrochloric acid was added and the mixture heated. After cooling, 10 mL of EDTA–Mg, 10 mL of anhydrous ethanol, 5 mL of buffer solution, and 4 drops of chromogenic black T were added, and titrated with 0.02 mol/L EDTA until the color changed from wine red to blue, recording the volume V_1_. A second 10 mL aliquot of the same solution was subjected to the same acidification and heating, with only 5 mL of buffer solution and 3 drops of indicator added, and titrated to the color change from purple to blue, recording the volume V_2_. Substituting V_1_ and V_2_ into Equation (1) yields *c* (SO_4_^2−^), and this procedure was repeated in parallel twice. The test results are shown in Table 3.

The microscopic analyses presented in this study primarily comprise scanning electron microscopy (SEM) and X-ray diffraction (XRD). The SEM was conducted with a Zeiss 300 instrument, and the XRD analysis was performed using a Malvern Panalytical X’Pert3 Powder diffractometer (by Malvern Panalytical B.V., Almelo, The Netherlands). The test setup is illustrated in Figure 4. SEM specimens were trimmed to maximum dimensions of 1 cm in length, width, and height. Due to the inherently poor electrical conductivity of concrete, the specimens were gold-sputtered to enhance conductivity and improve image clarity for better observation of microstructural features. For XRD testing, samples were taken from internal fragments after compression testing to minimize the influence of surface carbonization on the diffraction peaks of calcium carbonate. The samples were quickly vacuum-dried to further reduce carbonization effects.

## 3. Results and Analysis

### 3.1. Variations in Concrete Quality

**Figure 5:**
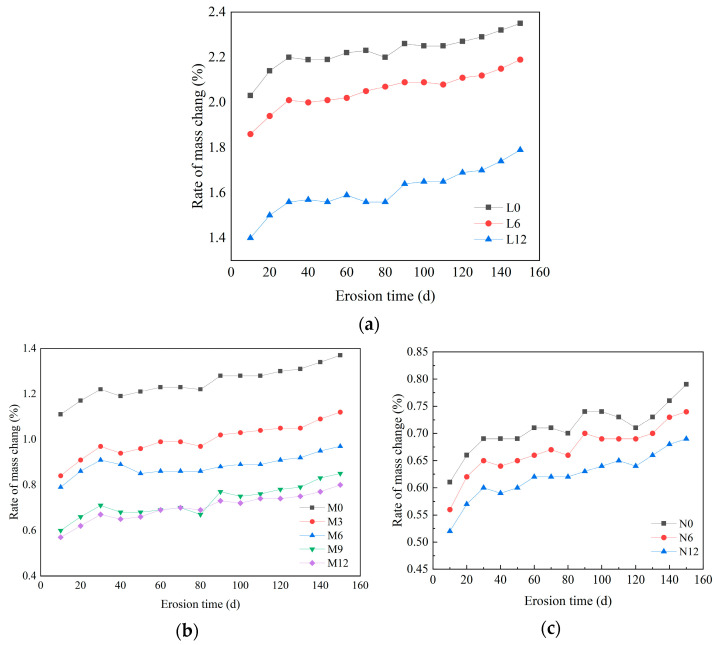
The mass change rate of concrete with different strength and different content of permeable crystallization materials. (**a**) C20, (**b**) C30, (**c**) C45.

Figure 5 presents the mass loss evolution of concrete specimens exposed to sulfate solution. The mass change rate follows a nonlinear trajectory, characterized by initial rapid growth that subsequently decelerates. Specimens incorporating CCCW exhibited substantially lower mass changes compared to the control group (0 kg/m^3^), with the reduction correlating positively with CCCW dosage. For C20 specimens (Figure 5a), the mass change rate of L12 decreased by 0.56% and 0.16% relative to L0 and L6, respectively. For C30 specimens (Figure 5b), M12 specimens demonstrated reductions of 0.54%, 0.49%, 0.34%, and 0.24% compared to M0, M3, M6, and M9, respectively. For C45 specimens (Figure 5c), N12 and N6 exhibited decreases of 0.09% and 0.04% relative to N0, respectively. These results demonstrate that increasing CCCW content effectively mitigates sulfate-induced mass changes and retards the formation of deleterious reaction products.

The enhanced sulfate resistance is primarily attributed to the penetration of active CCCW components into the concrete matrix, where they precipitate as crystalline products that densify the pore structure. Additionally, the crystallization process consumes calcium ions, reducing their availability for reaction with sulfate ions to form expansive products, thereby mitigating attack severity. Notably, for C30 specimens, the marginal improvement between M9 and M12 (0.49% versus 0.54% reduction relative to M0) suggests a plateau in performance enhancement at higher CCCW dosages. This diminishing return indicates that 9 kg/m^3^ represents an optimal dosage, balancing sulfate resistance performance with economic considerations.

### 3.2. Variation in Concrete Compressive Strength

**Figure 6:**
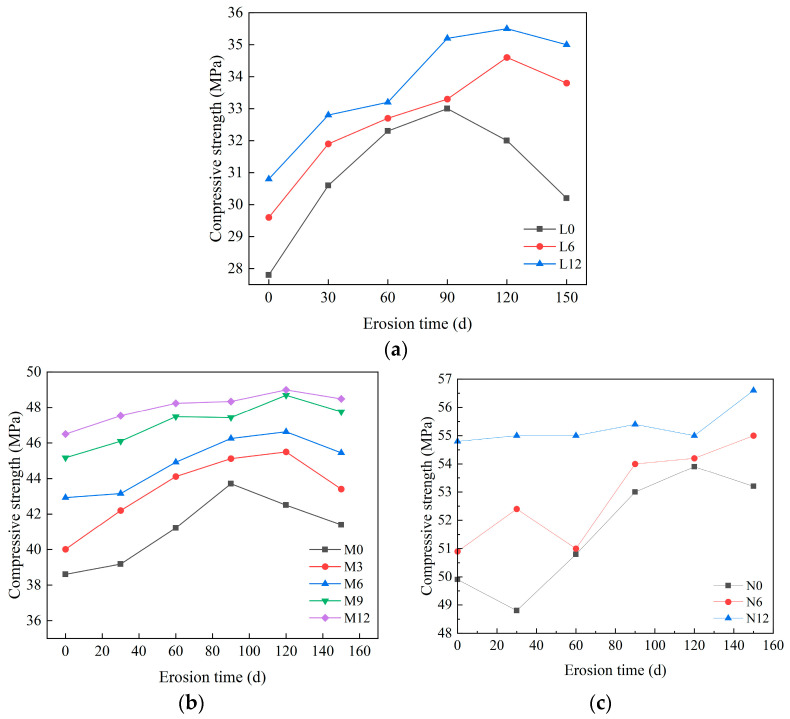
Compressive strength under different erosion time. (**a**) C20, (**b**) C30, (**c**) C45.

Figure 6 presents the evolution of compressive strength for concrete specimens under sulfate erosion, showing that CCCW content enhances strength and delays decay. In Figure 6a), initial strengths are 27.8 MPa (L0), 29.6 MPa (L6), and 30.8 MPa (L12), with overall strength rising initially and then declining as erosion progresses. L0 peaks around 90 days before decreasing due to sulfate attack forming expansive phases, whereas L6 and L12 maintain higher strengths with a more sustained rise, peaking at 120 days. This improvement is attributed to CCCW percolation and crystallization densifying the pore structure and delaying sulfate diffusion, as well as Ca^2+^ complexation reducing early expansive product formation. The maximum strength decline rates are 8.5% (L0), 2.3% (L6), and 1.4% (L12), indicating that CCCW markedly mitigates strength loss. Figure 6b,c show a similar trend. M0 peaks at 90 days, while CCCW-containing groups (M3–M12) peak at 120 days, with M12 reaching 49 MPa. Higher CCCW content further inhibits sulfate diffusion and slows expansive product formation via Ca^2+^ complexation, yielding lower strength loss (4.6% for M3, 2.6% for M6, 1.8% for M9, and 1.0% for M12) and a 98.2% strength retention at 150 days for M12 versus 94.7% for M0. In Figure 6c, the N0 specimen subjected to sodium sulfate immersion exhibits a compressive-strength trend that is similar to that of the other specimens. The observed decrease in the N0 specimen’s compressive strength at 30 days of immersion may be attributed to experimental variability. Therefore, increasing CCCW dosage promotes sustained strength growth and long-term retention in sulfate environment.

### 3.3. Variation in Ultrasonic Velocity

**Figure 7:**
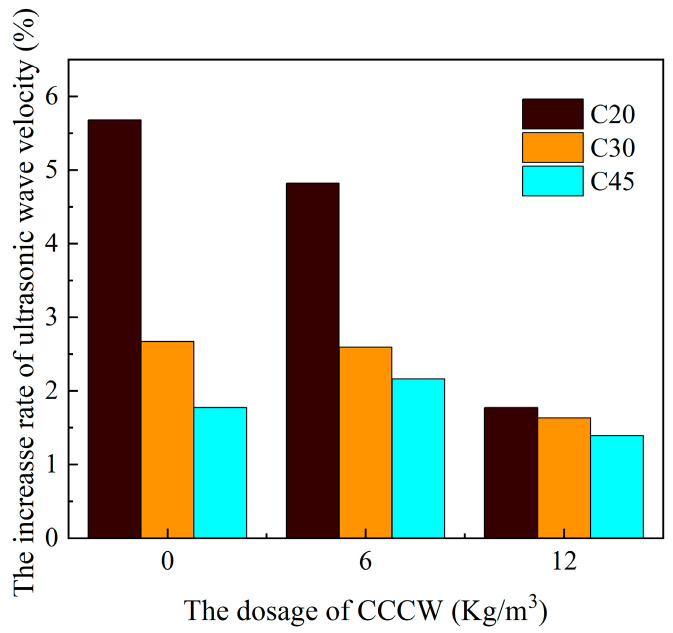
Variation in the ultrasonic lifting rate of concrete.

**Figure 8:**
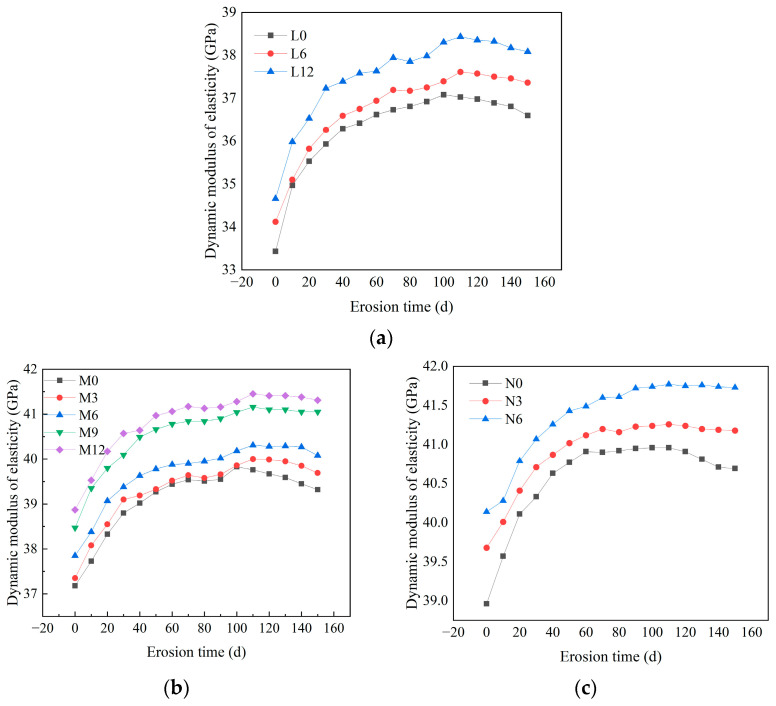
The dynamic elastic modulus of concrete. (**a**) C20, (**b**) C30, (**c**) C45.

Figure 7 shows the percentage increase in ultrasonic velocity from the initial ultrasonic velocity to the peak value for C20, C30, and C45 concrete. The percentage increase in ultrasonic velocity generally decreases with increasing concrete strength. For 0 kg/m^3^ CCCW concrete, the ultrasonic velocity increase gradually decreases with increasing concrete strength. When the concrete strength increases from C20 to C45, the ultrasonic velocity increase is 5.68%, 2.67%, and 1.77%, respectively. For 6 kg/m^3^ concrete, the ultrasonic velocity increase is 4.82%, 2.59%, and 2.16%, respectively, when the concrete strength increases from C20 to C45. For 12 kg/m^3^ concrete, the ultrasonic velocity increase is 1.77%, 1.63%, and 1.39%, respectively, when the concrete strength increases from C20 to C45. This is because the internal structure of high-strength concrete is relatively dense and has a lower porosity. This leaves less space for the CCCW material to fill the pores and improve the structure, thus limiting the improvement effect. Low-strength concrete has more internal pores, giving the CCCW material more space to penetrate and crystallize, filling the pores and significantly increasing the ultrasonic wave velocity. Figure 8 also shows that the L12, M12, and N12 groups had a low rate of increase in sound velocity throughout the immersion process, indicating a low rate of expansion product formation due to sulfate attack. Therefore, increasing concrete strength and increasing the dosage can both reduce the rate of increase in sound velocity and enhance its resistance to sulfate attack.

### 3.4. Variation in Dynamic Elastic Modulus of Concrete

Figure 8 illustrates the temporal evolution of dynamic elastic modulus in concrete under sulfate attack, exhibiting an initial rapid increase, followed by decelerated growth, peak attainment, and eventual decline. This pattern mirrors the progressive internal structural changes induced by corrosion. Comparisons across CCCW dosages (0, 6, and 12 kg/m^3^) reveal that untreated concrete displays a markedly lower initial modulus, underscoring CCCW’s efficacy in enhancing early stiffness. With prolonged exposure, CCCW-admixed concretes sustain elevated modulus levels, with the 12 kg/m^3^ variant consistently outperforming the 6 kg/m^3^ one, demonstrating dose-dependent improvements in stability and sulfate resistance. Mechanistically, CCCW promotes crystallization that fills pores and microcracks, densifying the matrix, boosting modulus, and impeding sulfate ingress to delay damage. Conversely, untreated concrete with higher porosity facilitates sulfate penetration and reactions with hydration products, accelerating structural degradation and modulus decline.

### 3.5. Characterization of Microstructure

**Figure 9:**
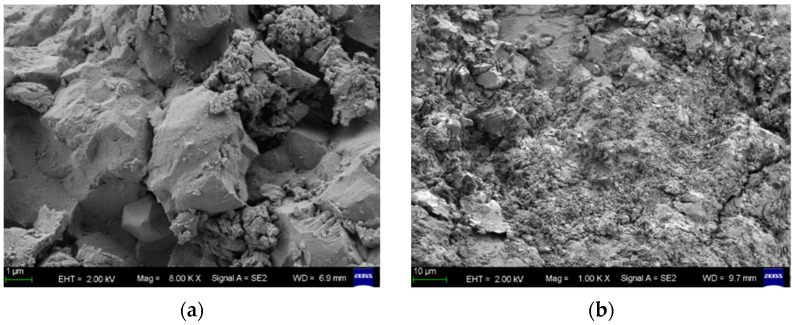
Microscopic appearance diagram of concrete without CCCW. (**a**) Before erosion, (**b**) after 150 days of erosion.

Figure 9 shows the microscopic appearance of C20 concrete without CCCW before and after erosion. Comparative analysis shows that the surface structure of the concrete specimen without CCCW is relatively loose, with numerous pores and microcracks, and the particles are not tightly connected. This provides penetration channels and reaction space for subsequent sulfate attack. In contrast, the concrete after 150 days of erosion undergoes significant sulfate attack, producing a large number of gypsum crystals. These gypsum crystals expand during their formation, causing damage to the concrete’s internal structure and the formation of microcracks. This indicates that sulfate has a strong corrosive effect on concrete, and the durability of concrete structures exposed to sulfate environments for a long time decreases with service life.

**Figure 10:**
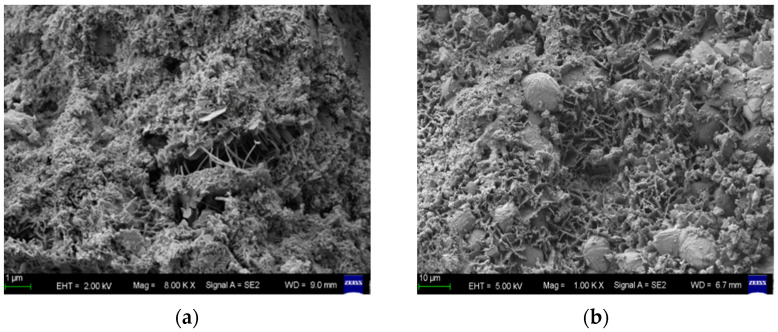
Microscopic appearance diagram of CCCW concrete. (**a**) Before erosion, (**b**) after 150 days of erosion.

Figure 10 presents the micromorphology of C20 concrete with a CCCW content of 12 kg/m^3^ before and after 150 days of sulfate exposure. Prior to exposure, the microstructure appears compact, with acicular crystals effectively bridging pores and microcracks, thereby impeding sulfate ion ingress and mitigating the formation of deleterious products such as gypsum. Post-exposure, the specimen exhibits minimal gypsum accumulation, accompanied by an extensive network of calcium silicate hydrate (C-S-H) gel that interconnects pores and fissures, resulting in subdued microcrack development. In comparison to Figure 9, CCCW incorporation yields a markedly denser microstructure, characterized by reduced porosity, enhanced matrix continuity, and more mature crystal formations. This enhancement stems from CCCW’s penetrative crystallization mechanism, wherein its active constituents react with cementitious hydration products (e.g., calcium ions) to generate insoluble crystals within capillary pores and microcracks. Such infilling not only seals microscopic voids but also establishes a protective barrier that curtails sulfate diffusion rates and depths, thereby attenuating reactions with hydration phases and suppressing expansive corrosion product formation. Consequently, CCCW admixture substantially bolsters concrete’s sulfate resistance and curtails the generation of harmful ettringite and gypsum phases.

### 3.6. X-Ray Diffraction Analysis

**Figure 11:**
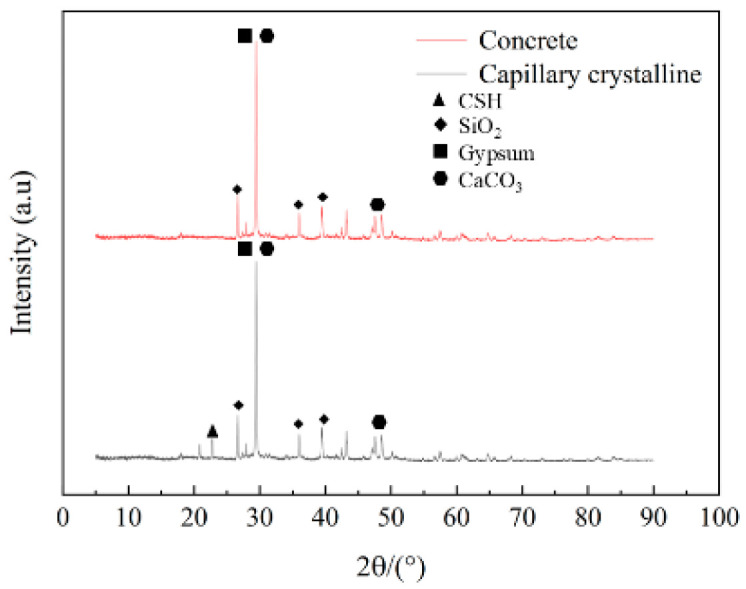
The XRD pattern of concrete.

Figure 11 shows the XRD results for concrete samples without CCCW and with 12 kg/m^3^ of CCCW after immersion in a sulfate solution for 150 days. Observing the XRD patterns, the relative positions of the two main diffraction peaks remain unchanged, indicating no new crystalline phase has formed. However, the intensities of the main diffraction peaks change significantly. Characteristic peaks of gypsum (CaSO_4_·2H_2_O) appear at 20.9°, 29.4°, and 60.0°, those of silica (SiO_2_) at 26.6° and 39.4°, and those of calcium carbonate (CaCO_3_) at 29.4°, 39.4°, 47.6°, and 48.6°. A further comparison of the XRD curves of sample L12 and sample L0 reveals that the characteristic SiO_2_ peak at 26.6° and the characteristic CaCO_3_ peak at 29.4° both show an upward trend. This indicates that with the incorporation of the CCCW, the active substances within it, such as silicates and soluble carbonates, react with the hydration products in the concrete to form gel-like water-insoluble calcium silicate hydrate gel (C-S-H), ettringite and insoluble CaCO_3_, which fill the concrete’s capillary pores. The generated C-S-H plugs microcracks, further improving concrete density and corrosion resistance. The C-S-H gel and CaCO_3_ fill the concrete’s capillary pores, forming a dense structure that blocks SO_4_^2−^ transport channels. Furthermore, the generation of C-S-H gel, ettringite and CaCO_3_ reduces the calcium source available for gypsum formation. And the reaction mechanism is shown in Equations (3) and (4). XRD analysis results show that the addition of infiltrating crystallization materials not only promotes the enrichment of calcium silicate hydrate and calcium carbonate, making the concrete structure denser, but also reduces the generation of sulfate attack products, thereby significantly improving the corrosion resistance of concrete in corrosive environments.

(3) $$CO_{3}^{2-}+Ca^{2+}\to CaCO_{3}↓$$

(4) $$SiO_{2}+Ca{\left(OH\right)}_{2}+\left(n-1\right)H_{2}O\to CaSiO_{3}\cdot nH_{2}O↓$$

**Figure 12:**
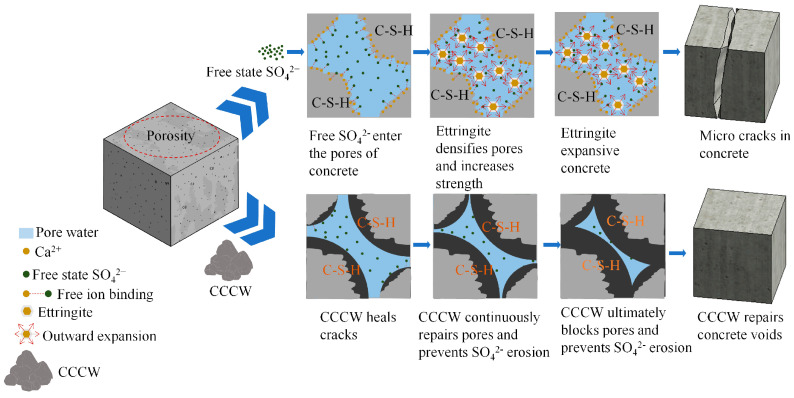
Sulfate attack and CCCW restoration.

Figure 12 illustrates the mechanism of sulfate ion attack on concrete and its repair using CCCW. Sulfate enters the pores of concrete and reacts with Ca^2+^ to form minerals such as ettringite. As the attack progresses, these minerals gradually accumulate and expand within the concrete pores, causing the concrete interior to expand. When the expansion volume exceeds the concrete’s capacity, microcracks initially form within the concrete, providing pathways for more sulfate to attack. These microcracks then gradually spread outward, eventually forming large cracks. Once large cracks form, more water or other corrosive ions enter the concrete, accelerating the attack, reducing its strength and durability, and in severe cases, even leading to structural failure. The addition of CCCW to concrete effectively reduces the initial porosity within the concrete, blocking the entry of sulfate ions. Furthermore, CCCW exhibits a self-healing effect on microcracks. Even if sulfate attacks the concrete, CCCW can repair the microcracks as it attacks. Ultimately, the pores and microcracks in the concrete are completely repaired, restoring its strength and improving its durability.

## 4. Diffusion of Sulfate Ions in Concrete

### 4.1. Sulfate Ion Concentration Distribution Characteristics

**Figure 13:**
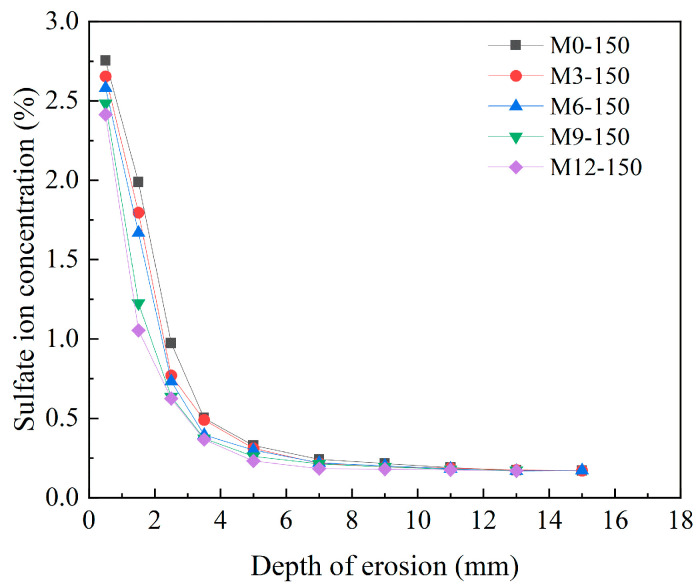
The distribution of sulfate ion concentration in concrete with different CCCW.

Figure 13 shows the variation in sulfate ion content with erosion depth in C30-strength concrete at different CCCW dosages after 150 days of erosion. A comparative analysis reveals that at 0.5 mm from the concrete surface, the sulfate ion concentration in specimens M0-150 is 2.75%, while that in specimens M3-M12 is 2.65%, 2.58%, 2.48%, and 2.41%, respectively. As the CCCW dosage increases, the sulfate ion content in the surface layer gradually decreases. This is due to the permeation crystallization of CCCW, which fills the capillary pores in the concrete and reduces the permeation channels for sulfate ions. A comparison of the maximum erosion depths of specimens with different dosages reveals that the maximum erosion depths for specimens with dosages of 0 kg/m^3^, 3 kg/m^3^, 6 kg/m^3^, 9 kg/m^3^, and 12 kg/m^3^ are 13 mm, 13 mm, 13 mm, 11 mm, and 11 mm, respectively. This shows that the incorporation of CCCW effectively slowed down the penetration of sulfate, and with the increase in CCCW dosage, the sulfate ion corrosion depth and sulfate ion content decreased significantly, indicating that CCCW has a certain inhibitory effect on sulfate corrosion.

**Figure 14:**
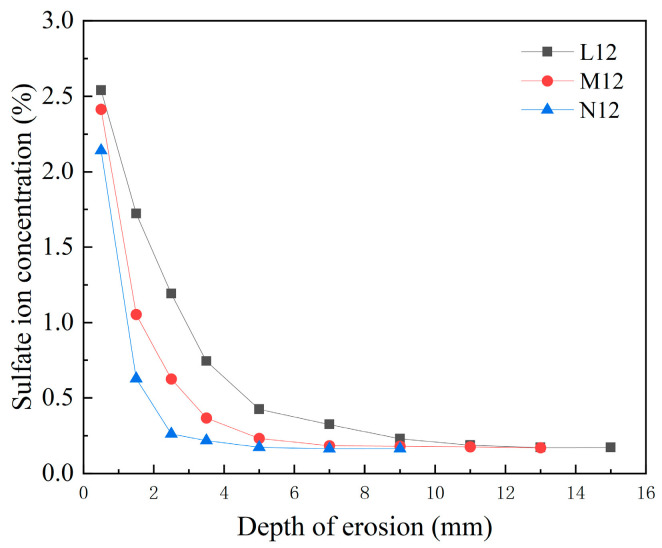
The distribution of sulfate ion concentration in 150 d concrete with different strength.

As shown in Figure 14, at the same CCCW dosage (12 kg/m^3^), the sulfate ion content and erosion depth of the C20, C30, and C45 concretes decreased significantly with increasing CCCW dosage. At an erosion depth of 0.5 mm, the sulfate ion content in the C20 specimen was 2.54%, while it decreased to 2.414% and 2.142% in the C30 and C45 specimens, respectively. At an erosion depth of 1.5 mm, the sulfate ion content in the C20 specimen was 1.959%, while it decreased to 1.054% and 0.627% in the C30 and C45 specimens, respectively. This indicates that increasing concrete strength can significantly reduce the sulfate ion content during sulfate attack. The maximum sulfate ion diffusion depth in the C20 specimen was 11 mm, while it decreased to 9 mm and 7 mm in the C30 and C45 specimens, respectively, indicating that increasing concrete strength significantly inhibits the longitudinal penetration of sulfate attack.

### 4.2. Determination of the Diffusion Coefficient of Sulfate Attack in Concrete

**Figure 15:**
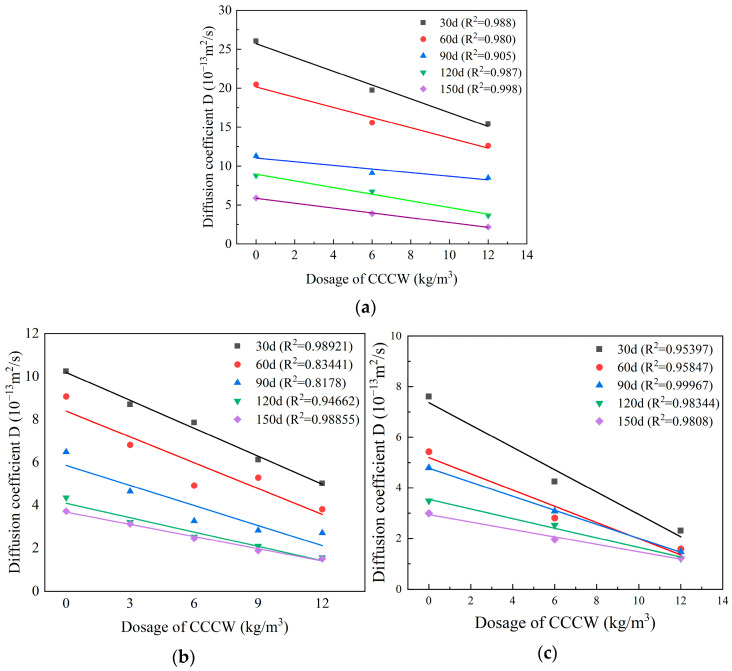
The relationship between the diffusion coefficient and the CCCW dosage under different erosion times. (**a**) C20, (**b**) C30, (**c**) C45.

Figure 15 shows the relationship between the diffusion coefficient and CCCW dosage at different erosion times. It can be seen that under different erosion cycles, the sulfate ion diffusion coefficient and CCCW dosage consistently exhibit a stable linear negative correlation. Statistical analysis of the experimental data reveals a high linear regression fit parameter, suggesting a linear function between the diffusion coefficient and dosage.

**Figure 16:**
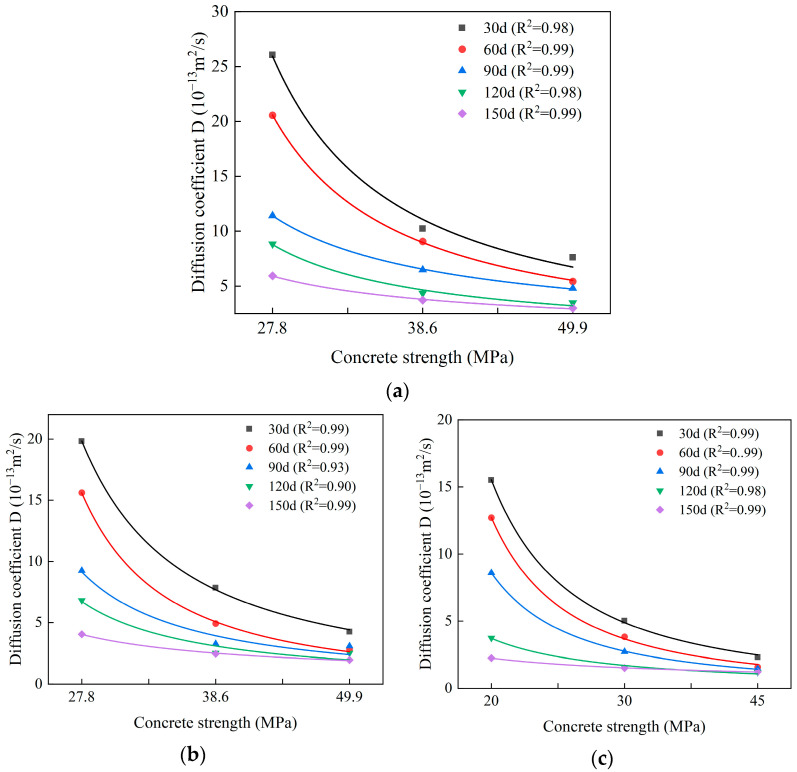
The relationship between the diffusion coefficient and the concrete strength under different erosion times. (**a**) 3 kg/m^3^, (**b**) 6 kg/m^3^, (**c**) 12 kg/m^3^.

Figure 16 delineates the correlation between sulfate ion diffusion coefficients and concrete strength grades across varying exposure durations. Notably, irrespective of erosion time or CCCW dosage, the diffusion coefficient diminishes with escalating concrete strength, exhibiting an initial sharp decline followed by asymptotic stabilization. Regression analysis employing a power function yielded excellent fits with high *R*^2^ values for all three CCCW dosages, indicative of a robust power-law dependency between sulfate diffusivity and concrete strength.

The sulfate diffusion coefficient D decreases with increasing CCCW dosage, concrete strength, and immersion time (Figure 16). Taking Figure 16a as an example, at a concrete strength of 27.8 MPa, D decreases significantly with longer immersion times: from 26.07 at 30 days to 5.94 at 150 days. The inverse relationship between the diffusion coefficient and immersion time indicates that, after six months of exposure, sulfates continue to damage microcracks in the concrete but are progressively repaired by CCCW, thereby reducing the diffusion capacity of sulfates within the concrete and demonstrating the long-term repair capability of CCCW for microcracks. Moreover, increasing the CCCW dosage markedly enhances this repair effect: at 150 days of exposure, the diffusion coefficients are D = 5.94, 4.05, and 2.24 for CCCW dosages of 3, 6, and 12 kg/m^3^, respectively, indicating a dose-dependent improvement in repair performance over the 150-day duration. When the concrete strength increases, the diffusion coefficient under the same immersion time decreases, which can be attributed to higher self-compaction, lower permeability, and diminished sulfate attack in stronger concrete. The in situ hydration of CCCW upon contact with water forms new hydrated calcium silicate phases that rapidly repair microcracks, thereby further lowering the sulfate diffusion coefficient.

The sulfate ion diffusion coefficient has a power function relationship with the erosion time and concrete strength, and a linear function relationship with the CCCW content. Therefore, the sulfate ion diffusion coefficient in an environment immersed in a 10% mass fraction Na_2_SO_4_ sodium sulfate solution can be expressed as Equation (5):

(5) $$D=a_{1}(b_{1}N+c_{1})\cdot (d_{1}\cdot M^{c_{1}})\cdot (f_{1}\cdot t^{g_{1}})$$

where *D* is the sulfate diffusion coefficient of concrete (10^−13^ m^2^/s), *N* is the CCCW dosage (kg/m^3^), *M* is the concrete strength (MPa), *t* is the erosion time (d), a_1_~g_1_ are the coefficients related to the Na_2_SO_4_ sulfate immersion environment and the test factors. The multivariate nonlinear least squares method was used to fit and optimize the test results, with an R^2^ of 0.89, and the diffusion coefficient equation can be expressed as:

(6) $$D=20.9768\times (-0.75N+19.2055)\times 21.0482M^{-2.0855}\times 21.0198t^{-0.7391}$$

**Figure 17:**
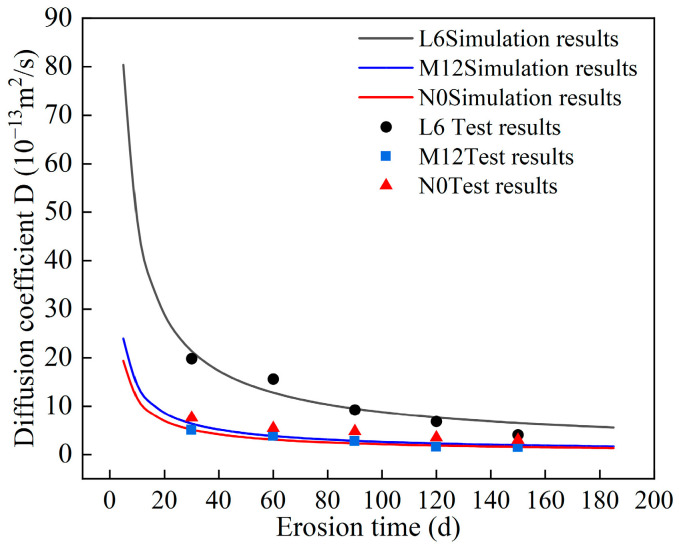
Simulation and test results of sulfate ion diffusion coefficient in concrete.

Diffusion coefficients for L6, M12, and N0 concretes across various exposure durations were computed using Equation (6) and benchmarked against empirical measurements. Figure 17 presents the simulated and experimental sulfate ion diffusion coefficients for diverse strength grades and CCCW dosages. The observed data points cluster tightly around the model-derived curve, underscoring the diffusion model’s high fidelity and predictive robustness.

## 5. Conclusions

This study investigates the sulfate erosion resistance of concrete admixed with CCCW and elucidates the effect of CCCW dosage on durability enhancement of different strength of concrete. The key findings are summarized as follows:

(1) CCCW significantly improves ultrasonic pulse velocity, and dynamic elastic modulus, and enhances long-term performance. Under sulfate attack, CCCW-admixed specimens exhibit higher dynamic elastic moduli with reduced declines.
(2) CCCW incorporation markedly enhances concrete density and sulfate resistance. It promotes increased formation of calcium silicate hydrate (C-S-H) through reactions with hydration products, leading to a denser pore structure and reduced generation of corrosion products.
(3) The diffusion coefficients decrease with increasing CCCW dosage and with higher concrete strength, albeit at a decelerating rate over time. The developed diffusion model provides a robust framework for evaluating sulfate resistance and guiding engineering optimizations.
(4) A reasonable amount of CCCW admixture can significantly improve the durability and mechanical stability of concrete in sulfate environments. In addition, a surface application of CCCW can also effectively reduce sulfate erosion damage, and the combined effect of internal addition and external spray of permeable crystallization waterproofing material on sulfate resistance of concrete requires further investigation.

## Figures and Tables

**Table 1:** Proportions of concrete.

Specimen Number	Strength	W/C	Cement (kg/m^3^)	Water (kg/m^3^)	Sand (kg/m^3^)	Coarse Aggregate (kg/m^3^)	CCCW(kg/m^3^)
L0	C20	0.79	267	211	769	1153	0
L6		0.79	267	211	769	1153	6
L12		0.79	267	211	769	1153	12
M0	C30	0.58	362	210	676	1152	0
M3		0.58	362	210	676	1152	3
M6		0.58	362	210	676	1152	6
M9		0.58	362	210	676	1152	9
M12		0.58	362	210	676	1152	12
N0	C45	0.45	467	210	594	1080	0
N6		0.45	467	210	594	1080	6
N12		0.45	467	210	594	1080	12

**Table 2:** Design of the number of concrete specimens.

Concrete Grade	CCCW (kg/m^3^)	Erosion Day (d)						Total
		0	30	60	90	120	150	
C20	0	3	3	3	3	3	3	18
	6	3	3	3	3	3	3	18
	12	3	3	3	3	3	3	18
C30	0	3	3	3	3	3	3	18
	3	3	3	3	3	3	3	18
	6	3	3	3	3	3	3	18
	9	3	3	3	3	3	3	18
	12	3	3	3	3	3	3	18
C45	0	3	3	3	3	3	3	18
	6	3	3	3	3	3	3	18
	12	3	3	3	3	3	3	18

**Table 3:** Test results.

Number	V_1_/mL	V_2_/mL	ΔV/mL	*c* (SO_4_^2−^)/mL	ε/%
1	7.57	0.01	7.56	0.00488	2.4
2	7.54	0.01	7.53	0.00494	1.2
3	7.55	0.01	7.54	0.00492	1.6

## Data Availability

The original contributions presented in this study are included in the article. Further inquiries can be directed to the corresponding author.
